# Small-scale risk assessment of transmission of parasites from wastewater treatment plant to downstream vegetable farms 

**Published:** 2018

**Authors:** Ehsan Javanmard, Hamed Mirjalali, Maryam Niyyati, Meysam Sharifdini, Esfandiar Jalilzadeh, Seyed Javad Seyed Tabaei, Hamid Asadzadeh Aghdaei, Roghieh Rostami, Ehsan Nazemalhosseini-Mojarad, Ali Haghighi, Mohammad Reza Zali

**Affiliations:** 1 *Department of Parasitology and Mycology, School of Medicine, Shahid Beheshti University of Medical Sciences, Tehran, Iran*; 2 *Foodborne and Waterborne Diseases Research Center, Research Institute for Gastroenterology and Liver Diseases, Shahid Beheshti University of Medical Sciences, Tehran, Iran.*; 3 *Department of Medical Parasitology and Mycology, School of Medicine, Guilan University of Medical Sciences, Rasht, Iran*; 4 *Department of Water and Wastewater Quality Control Laboratory, Water and Wastewater Company, Tehran, Iran*; 5 *Basic and Molecular Epidemiology of Gastrointestinal Disorders Research Center, Research Institute for Gastroenterology and Liver Diseases, Shahid Beheshti University of Medical Sciences, Tehran, Iran.*; 6 *Gastroenterology and Liver Diseases Research Center, Research Institute for Gastroenterology and Liver Diseases, Shahid Beheshti University of Medical Sciences, Tehran, Iran *

**Keywords:** Treated wastewaters, Vegetable farms, Irrigation, Parasitic contamination, Iran

## Abstract

**Aim:**

The aim of the present study was to simultaneously investigate parasitic contamination of treated wastewater and downstream vegetable farms that are irrigated with treated sewage, during a year.

**Background:**

(Oo) Cysts and eggs of parasites are resistant to most of routine wastewater treatment process. Irrigation of vegetables farms with either treated wastewater or illegally use of raw wastewaters enhances the risk of contamination with enteric pathogens.

**Methods:**

The treated wastewater samples were taken after chlorination from a wastewater treatment plant located at the south of Tehran. In addition, 60 vegetable samples (5 samples from each farm) were collected from the selected downstream farms that routinely used treated wastewater for irrigation of crops. Parasitological tests were performed using Ziehl–Neelsen, conventional lugol’s iodine staining and direct microscopical examination.

**Results:**

Parasites including free living larvae, eggs of *Toxoascaris leonina*, egg of *Toxocara* sp. *Trichuris* sp, *Trichostrongylus* sp and amoeboid trophozoite were seen in 5/12 (41.7%) of vegetable samples gathered during a year. There was no statistically significant correlation between the season and parasitic contamination of the vegetables (P= 1). Furthermore, parasitic contamination was observed in 7/12 (53.8%) of treated wastewater samples. The correlation between season and parasitic contamination of treated wastewater was evaluated that the results showed a higher contamination of treated wastewater in spring and autumn (P<0.05). Fisher’s exact test also showed that there was no significant correlation between parasitic contaminations of vegetable samples and treated wastewater according to seasonal change.

**Conclusion:**

The results showed parasites in both treated wastewater plant and downstream crops farms that suggests the public health importance of the quality of water resources that routinely used for irrigation of vegetable farms.

## Introduction

 Numerous beneficial effects of vegetables and fruits have convinced human populations to increase consumption of them. Due to the rich resources of fiber, protein, different kind of vitamins and vital mineral components, global demand for raw or lightly cooked vegetables and fruits have been dramatically increased (1-4). 

From the public health point of view, consumption of raw vegetables enhances the risk of microbial infections, particularly in those regions with traditional agriculture (5-7). Furthermore, limited water resources, global human population growth and beneficial effects of wastewater for farmland, the importance of the refined sewage for recreational uses and irrigation of agriculture farms have been emphasized (8-10). United Nations (UN) estimated that at least 50 countries routinely use treated or raw wastewater for irrigation of the farmlands (11). It seems that high microbial contamination of downstream farms reflects increased use of wastewater for irrigation of vegetables (12).

Foodborne parasites are considered as worldwide public health concern that can easily contaminate the farms that routinely use wastewater for irrigation of vegetables and crops (13, 14). Therefore, UN has targeted effective wastewater treatment to improve both chemical and microbial quality of treated sewage that is used for either recreational or irrigation goals in the Target 6.3 of Sustainable Development Goals (15). 

Although several studies have reported parasitic (protozoans, helminths) contamination of raw wastewater (16-18), there is not enough data about the parasitic pollution of treated wastewaters. According to the available data, although the great percent of microbial contaminations such as (oo) cysts and eggs of parasites are removed during treatment process, uncovered wastewater plant and draining canals can lead to secondary contamination due to access of animals (19, 20). On the other hand, this is well-documented that some (oo) cysts of parasites due to their tough wall are not totally eliminated during treatment process and thus would be concentrated in the treatment plant (21, 22). Therefore, remaining of pathogenic parasites during treatment process, secondary contamination of treated wastewaters (TW) resulted from animal access and also illegally irrigation of crops with raw sewage, increase the risk of contamination of the farmlands with parasites. The current study aimed to simultaneous investigate parasitic contamination of treated wastewater and downstream vegetable farms that are irrigated with treated sewage, during a year. 

## Methods

**Treated wastewater samples**

This was a cross‐sectional study that was performed during Sep 2016 to Aug 2017. TW samples were taken after chlorination from a wastewater treatment plant (WTP) located at the south of Tehran city. The TW produced by this WTP is used for irrigation of about 80000 hectares of downstream vegetable farms. This WTP uses settling of solid materials at the first step and further treatment using activated sludge and disinfection of the TW with chlorine. TW samples were collected from one meter below the water surface according to routine quality control and microbial analysis of the TW every month for a year and temperature as well as pH of the samples at the time of sampling were recorded. TW aseptically collected in 7-Liter container and after recording the time of sampling, pH, temperature and the level of resituate chlorine, samples were immediately transferred to Parasitology Lab of foodborne and waterborne Diseases Research Center, Research Institute for Gastroenterology and Liver Diseases, Shahid Beheshti University of Medical Sciences, Tehran, Iran for further analysis. 

**Vegetable samples**

**Figure 1:**
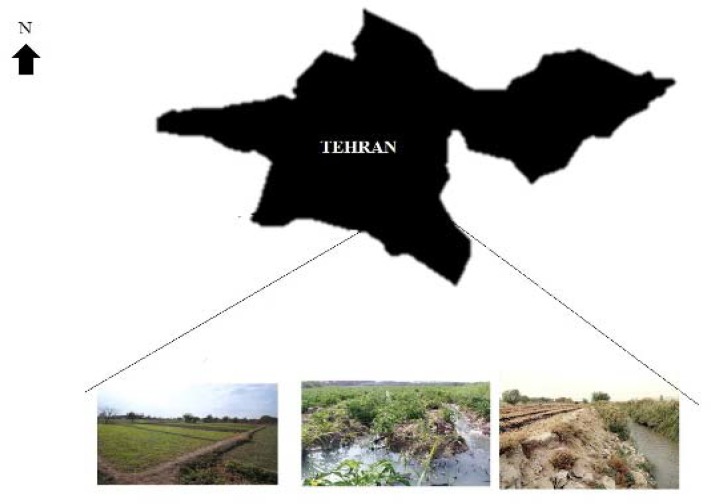
Schematic view of the sampling sites for vegetable and wastewater samples

Totally, 60 vegetable samples (5 samples from each farm) were collected from the selected farms near to the WTP that used TW for irrigation of crops (Figure 1). In order to reduce the chance of bias, a trained technician took different kind of vegetables including parsley, mint, basil, chives and dill from 5 sections (approximately 200-400 gram from each section) of each farm during 24 h after taking TW specimen. Samples were not examined according to the kind of vegetable and different sections of farm. Vegetables were collected in sterile polyethylene bags and immediately transferred to the laboratory for parasitological studies.

**Table 1:** Parasitic contamination of vegetable and TW samples according to season and month of the sampling

Sampling time		Treated wastewater		Vegetables	
		Helminthes (larvae/eggs)	Protozoa (cyst/trophozoite)	Helminthes (larvae/eggs)	Protozoa (cyst/trophozoite)
Autumn	September	Free-living larvae	-	-	-
	October	Free-living Larvae	-	Free-living Larvae	-
	November	Free-living larvae	Amoeboid trophozoite		-
Winter	December	-	-	Egg of *Trichuris *sp., Free-living larvae	-
	January	-	-	-	-
	February	-	-	-	-
Spring	March	Egg of *Toxocara* sp, Free-living larvae	-	Egg of *Trichostrongylus* sp.	Amoeboid trophozoite
	April	-	Amoeboid trophozoite		
	May	-	Amoeboid trophozoite	Egg of *Toxascaris leonine*., Free-living larvae	Amoeboid trophozoite
Summer	June	-	-	-	-
	July	-	-	-	-
	August	Egg of *Trichostrongylus *sp., Free-living larvae	-	Free-living larvae	-

**TW Filtration**

All the samples were aseptically filtered using Six-Branch filtration system with vacuum pump 24 L/sec (Sartorious, Goettingen, Germany). In order to filtration of TW, sterile 47-mm cellulose nitrate membrane (Sartorious, Goettingen, Germany) with pore size 0.4 µm was employed in each run. Furthermore, a 2-L of sterile water was used as negative control in each working run. Afterwards, the filtered membranes were completely rinsed in 30 mL of sterile PBS and kept out at 4-8 °C for more precipitation for 24 h. Then, the samples were centrifuged at 5000 rpm for 20min, supernatant was discarded and the pellet was examined for parasitological study. 

**Vegetable samples processing**

The total samples of each farm (approximately 1.5-2 kg) were weighted in 250 g portions and washed by 250 mL sterile PBS in a 2-L sterile container using vigorous agitation for 15 min. Then, the PBS rinsed materials were transferred to 2-L graduated cylinder and left for 24h at the room temperature. Afterwards, supernatant was discarded and the sediment was centrifuged at 5000 rpm for 20 min. Finally, supernatant was discarded and the pellet was introduced to the parasitology lab for further parasitological examination. 

Parasitological tests were performed using Ziehl–Neelsen, conventional lugol’s iodine staining and direct microscopical examination (×10 and ×40 objectives) for both vegetables and TW samples. 

## Results

Based on our findings, parasites were seen in 5/12 (41.7%) vegetable samples. Among the positive samples, free living larvae, eggs of *Toxoascaris leonina*, *Trichuris *sp, *Trichostrongylus* sp and amoeboid trophozoite were seen in 33.33%, 8.33%, 8.33%, 8.33% and 16.66%, respectively. Fisher’s Exact Test showed that although the number of contaminated vegetables in spring was more than other seasons, there was no statistically support for the correlation between season and parasitic contamination of the vegetables. 

From total of 12 TW samples, parasitic contamination was observed in 7/12 (53.8%) of the samples. Free living larvae (41.7%), egg of* Toxocara *sp (8.33%), egg of *Trichostrongylus* sp (8.33%) and amoeboid trophozoite (25%) were seen in parasite-positive TW samples. Based on the results of parasitological analysis of TW samples, most of the contamination were observed in spring and autumn while there was not seen parasitic contamination in winter. Statistical analysis using Fisher’s Exact Test represented significant correlation between season and parasitic contamination of TW (*P*<0.05). 

The statistical correlation between parasitic contaminations of vegetable samples with TW that was routinely used for irrigation of downstream farms was also investigated. Accordingly, Fisher’s Exact Test showed that there was no significant correlation between parasitic contaminations of vegetable and TW with respect to seasonal change. The relevant data is summarized in table 1.

Although the precise quantity of parasites in TW samples was not intended, the number of observed parasites after filtration of 7-liters of TW, sedimentation of filtered samples, and several slide preparation was very low.

## Discussion

 During the last decades, raw vegetables and fruits have been consumed as an important portion of dietary, all over the world (23). However, consumption of raw vegetables can increase the risk of gastrointestinal infections with broad spectrum of microbial agents including bacteria, viruses and parasites (6, 24-26). There are strong evidences signifying the role of raw and ready-to-eat vegetables in outbreaks, almost all over the world (27-30). However, it is well-established that illegally irrigation with raw wastewaters and even treated wastewater enhance the risk of contamination of downstream vegetables farms with enteric pathogens (8). Based on our best of knowledge, this is first study discussing simultaneous parasitic contamination of TW and downstream vegetable farms irrigated with TW according to the season. 

The current study showed 5/12 (41.7%) of vegetable samples were seen contaminated with at least one parasite (helminth and/or protozoan). Although the number of samples of the current study was significantly lower than other researches, the results are in line with previous studies in Iran. Accordingly, Shahnazi *et al *represented that 37.6% of unwashed vegetables were contaminated with parasites while there was not seen parasitic contamination in those vegetable samples which were washed before examination (31). After that, Fallah and his team investigated parasitic contamination of washed and unwashed salad vegetables and showed that the contamination of unwashed group was significantly higher than washed group (32). Highly contamination of vegetable samples was then reported by Ezatpour and colleagues that showed 52.7% contamination of the vegetable samples with parasitic microorganisms (33). Although the prevalence of parasitic contamination of vegetables in the current study was in line with the most of studies in Iran, the prevalence of the contamination was similar or even higher than the studies performed in other countries. Ali Mohamed and colleagues in Sudan showed that from 250 vegetable samples, 35 (13.5%) were contaminated with parasites (34). In another study in Turkey performed by Adanir *et al, *eggs of helminthic parasites were observed in 7/111 (6.3%) of raw vegetables that were randomly collected from bazaar (35). The lower prevalence of parasites in these studies compared with our study is probably related to the place of sampling. In other words, in our study vegetable samples were gathered from the farmland, but in these studies the examined vegetable samples were from center markets and probably after pre-washing. Another probable scenario for this finding could be related to the usage of night soil and illegally irrigation with raw wastewater in the farms that were included in our study. Moreover, almost all of these investigations were cross-sectional epidemiological studies and therefore the number of gathered samples were significantly higher than ours. In addition, the value of each vegetable sample in the current study was between 1 to 2 kg while the mentioned studies only assessed 200 gr of vegetable samples.

The possible correlation between the contaminations of vegetables with season was also evaluated and showed that although most of parasitic contaminations of vegetables were observed in spring, there was not statistical significant association between parasitic contamination and season. This result might be due to increase of rainfall in spring and the consequent runoff that can transport enteric pathogens excreted by animals to the downstream vegetables farms (36). 

As mentioned above, illegally irrigation of vegetable farms using raw wastewater as well as treated wastewaters running from uncovered canals can increase the possibility of microbial contaminations of downstream farms. Regarding this fact, parasitological analysis of treated wastewater showed contamination of 53.8% of TW samples with parasites. Couple of studies have indicated parasitic contamination of TW, all over the world. Ben Ayed and the colleagues showed that although sewage treatment eliminates a huge number of both parasitic helminths and protozoans from raw wastewater, the efficacy of this routine treatment process was not 100 percent (37). In the study practiced by Gupta *et al. *helminth eggs were recovered from 68.2% of TW (38). However, although the prevalence of parasitic contamination of TW of Gupta’s study is near to the findings of the current investigation, the mentioned study was cross-sectional and thus did not give annual view of parasitic contamination in a WTP. There is little data of parasitic contamination of TW in Iran. Mahvi and Kia examined raw and treated wastewater samples for helminth eggs and stated that in spite of presence of helminth eggs in TW samples, the mean number of the eggs per liter met WHO guidelines to reuse TW for vegetable irrigation (39). In the current study, most of the parasitic subjects in TW samples were attributed to non-pathogenic parasites. Furthermore, the number of each parasitic material was not calculated, but the current findings notify the importance of this water resources as a contamination source of vegetables farms.

Another outcome of this study was no statistical significant correlation between the presence of parasitic agents in TW and vegetable samples according to seasonal change, despite the higher contamination of both TW and vegetable samples in spring in comparison with other seasons. Indeed, quantity of parasites was not precisely calculated, but after concentration the samples using filtration and then centrifuging of the filtered samples, the number of parasitic materials was very low even after several slide preparations of each sediment. However, despite the elimination of huge number of the parasitic cysts/oocysts/eggs during treatment process, increased level of rainfall may enhance the probability of contamination of vegetables and WTPs. This fact happens via bringing the parasitic cysts/oocysts/eggs to the downstream farms and/or increasing the load of TW systems. However, remained parasitic materials are more likely at the range of WHO guidelines for irrigation of vegetable farms (40). 

Nevertheless, simultaneous presence of parasitic materials in TW samples as well as vegetable farms, which were irrigated with TW, indicates the importance of regular periodic quality control of water treatment process for parasitic contamination even though the number of the parasitic materials in WTPs be at the range of WHO guidelines. 

Results of the current study indicated that it was seen parasitic contamination in both treated wastewaters and raw vegetables, which were irrigated with treated wastewaters. This finding suggests the importance of water resources that are applied for irrigation of vegetables, particularly in case of parasites that can remain infective during treatment process and transmit to downstream vegetable farms. 

## References

[1] Su LJ, Arab L (2006). Salad and raw vegetable consumption and nutritional status in the adult US population: results from the Third National Health and Nutrition Examination Survey. J Am Diet Assoc.

[2] Taremi M, Soltan Dallal M, Gachkar L, MoezArdalan S, Zolfagharian K, Zali MR (2006). Prevalence and antimicrobial resistance of Campylobacter isolated from retail raw chicken and beef meat, Tehran, Iran. Int J Food Microbiol..

[3] Liu X, Yan Y, Li F, Zhang D (2016). Fruit and vegetable consumption and the risk of depression: A meta-analysis. Nutrition.

[4] Wu QJ, Wu L, Zheng LQ, Xu X, Ji C, Gong TT (2016). Consumption of fruit and vegetables reduces risk of pancreatic cancer: evidence from epidemiological studies. Eur J Cancer Prev.

[5] Hou Z, Fink RC, Radtke C, Sadowsky MJ, Diez-Gonzalez F (2013). Incidence of naturally internalized bacteria in lettuce leaves. Int J Food Microbiol.

[6] Park S, Szonyi B, Gautam R, Nightingale K, Anciso J, Ivanek R (2012). Risk factors for microbial contamination in fruits and vegetables at the preharvest level: a systematic review. J Food Protect.

[7] Taremi M, Khoshbaten M, Gachkar L, EhsaniArdakani M, Zali M (2005). Hepatitis E virus infection in hemodialysis patients: a seroepidemiological survey in Iran. BMC Infect Dis.

[8] Jiménez B (2006). Irrigation in developing countries using wastewater. Inter Rev Environ Strategies.

[9] Wichelns D, Drechsel P, Qadir M (2015). Wastewater: Economic asset in an urbanizing world.

[10] Evans AEV, Hanjra MA, Jiang YL, Qadir M, Drechsel P (2012). Water Quality: Assessment of the Current Situation in Asia. Int J Water Resour D.

[11] United Nations (2003). Water for people, water for life The United Nations world water development report.

[12] Mara D, Sleigh A (2010). Estimation of norovirus and Ascaris infection risks to urban farmers in developing countries using wastewater for crop irrigation. J Water Health..

[13] Anuar TS, Salleh FM, Moktar N (2014). Soil-transmitted helminth infections and associated risk factors in three Orang Asli tribes in Peninsular Malaysia. Sci Rep.

[14] Pham-Duc P, Nguyen-Viet H, Hattendorf J, Zinsstag J, Phung-Dac C, Zurbrugg C (2013). Ascaris lumbricoides and Trichuris trichiura infections associated with wastewater and human excreta use in agriculture in Vietnam. Parasitol Int.

[15] United Nations (2015). Water and Sanitation the Pathway to a Sustainable Future.

[16] Hatam Nahavandi K, Mahvi AH, Mohebali M, Keshavarz H, Rezaei S, Mirjalali H (2016). Molecular Typing of Eimeria ahsata and E crandallis Isolated From Slaughterhouse Wastewater. Jundishapur J Microbiol.

[17] Hatam-Nahavandi K, Mohebali M, Mahvi AH, Keshavarz H, Najafian HR, Mirjalali H (2016). Microscopic and Molecular Detection of Cryptosporidium andersoni and Cryptosporidium xiaoi in Wastewater Samples of Tehran Province, Iran. Iran J Parasitol.

[18] Hatam-Nahavandi K, Mohebali M, Mahvi AH, Keshavarz H, Mirjalali H, Rezaei S (2017). Subtype analysis of Giardia duodenalis isolates from municipal and domestic raw wastewaters in Iran. Environ Sci Pollut Res Int.

[19] Lonigro A, Rubino P, Lacasella V, Montemurro N (2016). Faecal pollution on vegetables and soil drip irrigated with treated municipal wastewaters. Agric Water Manag.

[20] Kuroda K, Nakada N, Hanamoto S, Inaba M, Katayama H, Do AT (2015). Pepper mild mottle virus as an indicator and a tracer of fecal pollution in water environments: comparative evaluation with wastewater-tracer pharmaceuticals in Hanoi, Vietnam. Sci Total Environ.

[21] Forslund A, Plauborg F, Andersen MN, Markussen B, Dalsgaard A (2011). Leaching of human pathogens in repacked soil lysimeters and contamination of potato tubers under subsurface drip irrigation in Denmark. Water Res.

[22] Burnet JB, Penny C, Ogorzaly L, Cauchie HM (2014). Spatial and temporal distribution of Cryptosporidium and Giardia in a drinking water resource: implications for monitoring and risk assessment. Sci Total Environ.

[23] Hobbs SH (2005). Attitudes, practices, and beliefs of individuals consuming a raw foods diet. Explore (New York, NY).

[24] Losio MN, Pavoni E, Bilei S, Bertasi B, Bove D, Capuano F (2015). Microbiological survey of raw and ready-to-eat leafy green vegetables marketed in Italy. Int J Food Microbiol.

[25] Balkhair KS (2016). Microbial contamination of vegetable crop and soil profile in arid regions under controlled application of domestic wastewater. Saudi J Biol Sci..

[26] Gomez-Govea M, Solis-Soto L, Heredia N, Garcia S, Moreno G, Tovar O (2012). Analysis of microbial contamination levels of fruits and vegetables at retail in Monterrey, Mexico. J Food Agric Environ.

[27] Drankin DI, Zaiats NA, Krylov BA, Reshetnikova VI, Riabinin NV, Kharitonova LP (1995). [A water-borne outbreak of hepatitis A among students employed in agricultural operations on a vegetable-growing farm]. Zh Mikrobiol Epidemiol Immunobiol.

[28] Jernberg C, Hjertqvist M, Sundborger C, Castro E, Lofdahl M, Paajarvi A (2015). Outbreak of Salmonella Enteritidis phage type 13a infection in Sweden linked to imported dried-vegetable spice mixes, December 2014 to July 2015. Euro Surveill.

[29] Jung Y, Jang H, Matthews KR (2014). Effect of the food production chain from farm practices to vegetable processing on outbreak incidence. Microb Biotechnol.

[30] Karanis P, Kourenti C, Smith H (2007). Waterborne transmission of protozoan parasites: a worldwide review of outbreaks and lessons learnt. J Water Health.

[31] Shahnazi M, Jafari-Sabet M (2010). Prevalence of Parasitic Contamination of Raw Vegetables in Villages of Qazvin Province, Iran. Foodborne Pathog Dis.

[32] Fallah AA, Pirali-Kheirabadi K, Shirvani F, Saei-Dehkordi SS (2012). Prevalence of parasitic contamination in vegetables used for raw consumption in Shahrekord, Iran: Influence of season and washing procedure. Food Control.

[33] Ezatpour B, Chegeni AS, Abdollahpour F, Aazami M, Alirezaei M (2013). Prevalence of parasitic contamination of raw vegetables in Khorramabad, Iran. Food Control.

[34] Mohamed MA, Siddig EE, Elaagip AH, Edris AMM, Nasr AA (2016). Parasitic contamination of fresh vegetables sold at central markets in Khartoum state, Sudan. Ann Clin Microb Anti.

[35] Adanir R, Tasci F (2013). Prevalence of helminth eggs in raw vegetables consumed in Burdur, Turkey. Food Control.

[36] Daryani A, Ettehad G, Sharif M, Ghorbani L, Ziaei H (2008). Prevalence of intestinal parasites in vegetables consumed in Ardabil, Iran. Food control.

[37] Ben Ayed L, Schijven J, Alouini Z, Jemli M, Sabbahi S (2009). Presence of parasitic protozoa and helminth in sewage and efficiency of sewage treatment in Tunisia. Parasitol Res.

[38] Gupta N, Khan DK, Santra SC (2009). Prevalence of intestinal helminth eggs on vegetables grown in wastewater-irrigated areas of Titagarh, West Bengal, India. Food Control.

[39] Mahvi AH, Kia EB (2006). Helminth eggs in raw and treated wastewater in the Islamic Republic of Iran. East Mediterr Health J.

[40] World Health Organization ( 2006). Guidelines for the safe use of wastewater, excreta and greywater.

